# Identification of amino acid residues in the ligand binding repeats of LDL receptor important for PCSK9 binding[S]

**DOI:** 10.1194/jlr.M089193

**Published:** 2019-01-07

**Authors:** Shi-jun Deng, Adekunle Alabi, Hong-mei Gu, Ayinuer Adijiang, Shucun Qin, Da-wei Zhang

**Affiliations:** Department of Pediatrics, Group on the Molecular and Cell Biology of Lipids,* University of Alberta, Edmonton, Alberta, Canada; Department of Biochemistry, Faculty of Medicine and Dentistry,† University of Alberta, Edmonton, Alberta, Canada; Institute of Atherosclerosis,§ Taishan Medical University, Taian, China

**Keywords:** LDL binding, hypercholesterolemia, atherosclerosis, site-directed mutagenesis

## Abstract

Proprotein convertase subtilisin/kexin type 9 (PCSK9) promotes LDL receptor (LDLR) degradation, increasing plasma levels of LDL cholesterol and the risk of cardiovascular disease. We have previously shown that, in addition to the epidermal growth factor precursor homology repeat-A of LDLR, at least three ligand-binding repeats (LRs) of LDLR are required for PCSK9-promoted LDLR degradation. However, how exactly the LRs contribute to PCSK9’s action on the receptor is not completely understood. Here, we found that substitution of Asp at position 172 in the linker between the LR4 and LR5 of full-length LDLR with Asn (D172N) reduced PCSK9 binding at pH 7.4 (mimic cell surface), but not at pH 6.0 (mimic endosomal environment). On the other hand, mutation of Asp at position 203 in the LR5 of full-length LDLR to Asn (D203N) significantly reduced PCSK9 binding at both pH 7.4 and pH 6.0. D203N also significantly reduced the ability of LDLR to mediate cellular LDL uptake, whereas D172N had no detectable effect. These findings indicate that amino acid residues in the LRs of LDLR play an important role in PCSK9 binding to the receptor.

Plasma levels of cholesterol, especially LDL cholesterol (LDL-C), are positively correlated with the risk of atherosclerosis (1, 2). The LDL receptor (LDLR)-mediated LDL uptake plays an essential role in the clearance of plasma LDL-C (3). Upon LDL binding to the N-terminal ligand-binding repeats (LRs) of LDLR, the receptor and LDL complex are internalized via clathrin-coated pits and delivered to endosomes, where LDL is released from the receptor and delivered to lysosomes for degradation, LDLR is then recycled to the cell surface (2, 4, 5). Mutations in LDLR cause familial hypercholesterolemia (FH) and increase the risk for atherosclerotic coronary heart disease (2, 3). *Ldlr^−/−^* mice display higher plasma levels of cholesterol, especially LDL-C, than WT littermates and develop atherosclerosis when fed a high-cholesterol diet (6).

Proprotein convertase subtilisin/kexin type 9 (PCSK9) is a 692 amino acid secreted glycoprotein that consists of a 30 amino acid signal sequence followed by a prodomain, a catalytic domain, and a C-terminal domain. Expression of PCSK9 is high in the liver, intestine, kidney, and brain (7, 8). PCSK9 binds to LDLR and redirects the receptor for lysosomal degradation (8–14), playing a central role in regulating plasma LDL-C. Gain-of-function mutations in PCSK9 lead to elevated plasma LDL-C levels and accelerated atherosclerosis and premature coronary heart disease (15). Conversely, loss-of-function PCSK9 mutations lead to reduced plasma LDL-C levels and protection from coronary heart disease (16). Increased plasma levels of PCSK9 in mice preferentially promote LDLR degradation in the liver, but not in other tissues (17). We and others have shown that PCSK9 interacts with the epidermal growth factor precursor homology repeat-A (EGF-A) of LDLR at the cell surface and binds to the receptor with a much higher affinity at the acidic environment of the endosome (11, 18, 19). Consequently, the receptor is redirected from the endosome to the lysosome for degradation, rather than being recycled (11). The X-ray crystallographic structure of PCSK9 with the partial extracellular domain of LDLR at a neutral pH value shows that the EGF-A and YWTD repeats of LDLR interact with the catalytic domain and the prodomain of PCSK9, respectively (20). However, the LR1 to LR6 of LDLR are absent in the structure. We have demonstrated that, in addition to the EGF-A and YWTD repeats, a minimum of three LRs in LDLR are essential for efficient LDLR degradation induced by PCSK9 (12, 13). Several biochemical studies indicate that the negatively charged LRs of LDLR may interact with the positively charged C-terminal domain of PCSK9 at the cell surface and/or in the acidic endosomal environment to enhance PCSK9 binding (21–23). To further investigate the role of the LRs of LDLR in PCSK9-promoted LDLR degradation, we replaced negatively charged residues in the LRs of LDLR and assessed the effects of these mutations on PCSK9 binding. The numbers that indicated the positions of amino acid residues in LDLR in this study were counted from the N terminus of the receptor without the 21 amino acid signal sequence. We found that Asp172 in the linker and Asp203 in the LR5 of LDLR played a role in PCSK9 binding.

## MATERIALS AND METHODS

### Materials

DMEM, FBS, penicillin-streptomycin, trypsin-EDTA solution, Dil-labeled human LDL, and unlabeled human LDL were obtained from ThermoFisher Scientific. Complete EDTA-free protease inhibitors and X-tremeGENE^TM^ HP DNA transfection reagent were purchased from Millipore Sigma. Peptide-*N*-glycosidase F (PNGase F) was obtained from New England Biolabs (Beverly, MA). All other reagents were obtained from Fisher Scientific, unless otherwise indicated.

### Site-directed mutagenesis and immunoblot analysis

Plasmid pcDNA3.1 or pBud (ThermoFisher Scientific) containing cDNA of the full-length WT LDLR or the deletion mutant (LDLR-ΔLR4-LR7) was used to generate the mutant forms of LDLR. Mutagenesis was performed using QuikChange lightning site-directed mutagenesis kits (Agilent Technologies) as described in our previous studies (13, 24–27). The sequences of the oligonucleotides containing the residues to be mutated were synthesized by IDT, Inc. (Coralville, IA). The presence of the desired mutation and the integrity of each construct were verified by DNA sequencing.

Cells were collected and lysed in the lysis buffer (1% Triton, 150 mM NaCl, and 50 mM HEPES, pH 7.4) containing Complete EDTA-free protease inhibitors for 30 min on ice. After centrifugation for 10 min at 20,000 *g* at 4°C, the supernatant was collected, and protein concentrations were determined by the BCA protein assay. The same amount of cell lysate proteins was subjected to SDS-PAGE (8%) and transferred to nitrocellulose membranes (GE Healthcare) by electroblotting. Immunoblotting was performed using specific Abs as indicated. Ab binding was detected using IRDye680- or IRDye800-labeled goat anti-mouse or anti-rabbit IgG (Li-Cor). The signals were detected on a Li-Cor Odyssey Infrared Imaging System (Li-Cor).

### Binding of PCSK9 to LDLR and PCSK9-promoted LDLR degradation

The experiments were performed as described in our previous studies (11–13). Basically, the recombinant full-length human PCSK9 containing a FLAG tag at the C terminus was purified from HEK 293S cells as described (11–13). HEK293 cells were maintained in DMEM containing 10% FBS at 37°C in a 5% CO_2_ humidified incubator. Cells were seeded in 12-well dishes in 1 ml of culture medium containing 1.5 × 10^5^ cells/well. At 24 h later, the cells were transfected with empty plasmid or a plasmid carrying the WT or mutant LDLR cDNA using X-tremeGENE HP (1.0 μg of DNA and 2.5 μl of HP per well), according to the manufacturer’s protocol. For PCSK9 binding, 48 h after transfection, binding of PCSK9 to LDLR at different pH values was carried out as described (13). Briefly, cells were washed twice with ice-cold pH 7.4 buffer (50 mM Tris-HCl, 150 mM NaCl, 2 mM CaCl_2_, and 2.5% nonfat milk) or pH 6.0 buffer (25 mM Tris-Maleate buffer, 150 mM NaCl, 2 mM CaCl_2_, and 2.5% nonfat milk) and chilled on ice for 30 min in the pH buffer to inhibit LDLR endocytosis. PCSK9 (2 μg/well) was then added to the chilled cells in the ice-cold relevant pH buffer and incubated for 2 h on ice that could inhibit PCSK9-promoted LDLR degradation. Afterward, the cells were washed twice in the ice-cold relevant pH buffer and then collected for the preparation of whole-cell lysate. For PCSK9-promoted LDLR degradation, 48 h after transfection, the cells were washed twice in DMEM and then incubated with DMEM containing different amounts of PCSK9 as indicated for 12 h at 37°C in a 5% CO_2_ humidified incubator. The cells were then washed twice in PBS and collected for the preparation of whole-cell lysate. Protein concentrations were determined by the BCA protein assay. The same amount of whole-cell lysate was subjected to SDS-PAGE (8%) and immunoblotted using the following Abs: 15A6, a monoclonal anti-PCSK9 Ab; 13D3, a monoclonal anti- catalytic domain of PCSK9 Ab (12); 9E10, a monoclonal anti-Myc tag Ab; HL-1, a monoclonal anti-LDLR Ab (11, 12); a monoclonal anti-actin Ab; and a polyclonal anti-calnexin Ab (Invitrogen).

### Dil-LDL uptake by LDLR

Hepa1c1c7 cells were seeded at a density of 30,000 cells/well in a 96-well plate in 100 μl of MEM-α containing 10% FBS. At 24 h later, the cells were transfected with a plasmid carrying the WT or mutant LDLR cDNA using X-tremeGENE HP (0.1 μg of DNA and 0.25 μl of HP per well), according to the manufacturer’s protocol. At 48 h after transfection, the LDL binding assay was performed as previously described (28, 29). Briefly, cells were washed with Opti-MEM. Dil-labeled LDL (10 μg/ml) was then added to cells in 100 μl of MEM-α containing 5% newborn calf lipoprotein-poor serum in the presence or absence of unlabeled human LDL (600 μg/ml). The plates were incubated at 37°C for 6 h. Afterward, the cells were washed four times in the washing buffer (50 mM Tris-HCl, pH 7.4, 150 mM NaCl, and 2 mg/ml BSA) and then lysed in 100 μl of RIPA buffer (50 mM Tris-HCl, pH 7.4, 150 mM NaCl, 1% Triton X-100, 0.5% sodium deoxycholate, and 0.1% SDS) containing protease inhibitors. The lysate was then transferred to a 96-well black plate for the measurement of fluorescence using a SYNERGY plate reader at an excitation wavelength of 520 nm and an emission wavelength of 580 nm. The concentrations of total proteins in each well were measured using the BCA protein assay. LDL uptake was calculated by normalization of the fluorescence units to the amount of total proteins in the same well. The results obtained in the presence of excess unlabeled LDL revealed the nonspecific binding. Specific binding was calculated by subtraction of nonspecific binding from the total counts measured in the absence of unlabeled LDL.

### Biotinylation of cell surface proteins

HEK293 cells were seeded in a 6-well plate in 2 ml of culture medium at the density of 2.5 × 10^5^ cells/well. At 24 h later, cells were transfected with empty plasmid or a plasmid carrying the WT or mutant LDLR cDNA using X-tremeGENE HP (2 μg DNA and 5 μl HP/well). At 48 h after transfection, cell-surface proteins were biotinylated exactly as previously described (30). Briefly, cells were incubated with 0.5 mg/ml EZ-Link Sulfo-NHS-LC-Biotin (Pierce) in PBS (pH 8.0) for 15 min at 4°C. After quenching with 100 mM glycine, the cells were lysed in 150 μl of the lysis buffer. A total of 50 μl of the lysate was saved, and approximately 100 μl of the lysate (same amount of total proteins) was added to 60 μl of 50% slurry of Neutravidin agarose (Pierce) to pull down biotinylated cell-surface proteins. The cell-surface proteins were eluted from the beads by adding 1× SDS loading buffer (31 mM Tris-HCl, pH 6.8, 1% SDS, 12.5% glycerol, and 0.0025% bromophenol blue) and then analyzed by SDS-PAGE and immunoblotting.

### Deglycosylation of LDLR

HEK293 cells transiently expressing the WT or mutant LDLR were washed twice with PBS and collected in 1 ml of ice-cold PBS containing 2 mM EDTA. After centrifugation at 20,000 *g* for 2 min at 4°C, the cell pellets were resuspended in 20 μl of 1× glycoprotein denaturing buffer, gently mixed, heated at 100°C for 10 min, and then chilled on ice for 10 s. Afterward, 4 μl of 10× G7 reaction buffer (New England Biolabs), 4 μl of 10% Nonidet P-40 (vol/vol), and 12 μl of H_2_O were added to the sample. Each sample was then supplied with 2 μl of PNGase F or H_2_O, followed by incubation at 37°C for 1 h. Protein glycosylation was monitored by immunoblotting.

### Inhibition of *N*-glycosylation

HEK293 cells seeded in a 12-well plate were transfected with empty plasmid or a plasmid carrying the WT or mutant LDLR cDNA using X-tremeGENE HP (1 μg of DNA and 2.5 μl of HP per well). At 24 h later, the cells were incubated with tunicamycin dissolved in DMSO (Sigma, 0.5 μg/ml) or DMSO at 37°C for 24 h. The cells were then washed twice in PBS and collected for the preparation of whole-cell lysate and immunoblotting.

### Immunofluorescence of LDLR

Confocal microscopy was carried out as described previously (13, 25, 30). Briefly, HEK293 cells seeded onto the coverslips (1.0 × 10^5^ cells/ml) were transfected with the WT or mutant LDLR cDNA as indicated. At 48 h later, cells were fixed with 4% paraformaldehyde and permeabilized with cold methanol. After blocking with 1% BSA, the cells were incubated with an anti-LDLR monoclonal Ab and an anti-Na^+^/K^+^-ATPase polyclonal Ab (1:200) (Abcam). Ab binding was detected using Alexa Fluor 488 goat anti-rabbit IgG and Alexa Fluor 568 goat anti-mouse IgG. After washing, coverslips were mounted on the slides with Antifade reagent containing 4′,6-diamidino-2-phenylindole (DAPI) (Vector Laboratories, Burlingame, CA). Localizations of LDLR and Na^+^/K^+^-ATPase in the transfected cells were determined using a Leica SP5 laser-scanning confocal microscope (filters: 461 nm for DAPI, 519 nm for Fluor 488, and 603 nm for Fluor 568).

### Statistical analysis

All statistical analyses were carried out by GraphPad Prism version 4.0 (GraphPad Software). Student’s *t*-test was used to determine the significant differences between groups. The significance was defined as *P* < 0.05. Results are presented as mean ± SD.

## RESULTS

### Effect of mutations in LDLR-ΔLR4-LR7 on PCSK9 binding

**Fig. 1.**
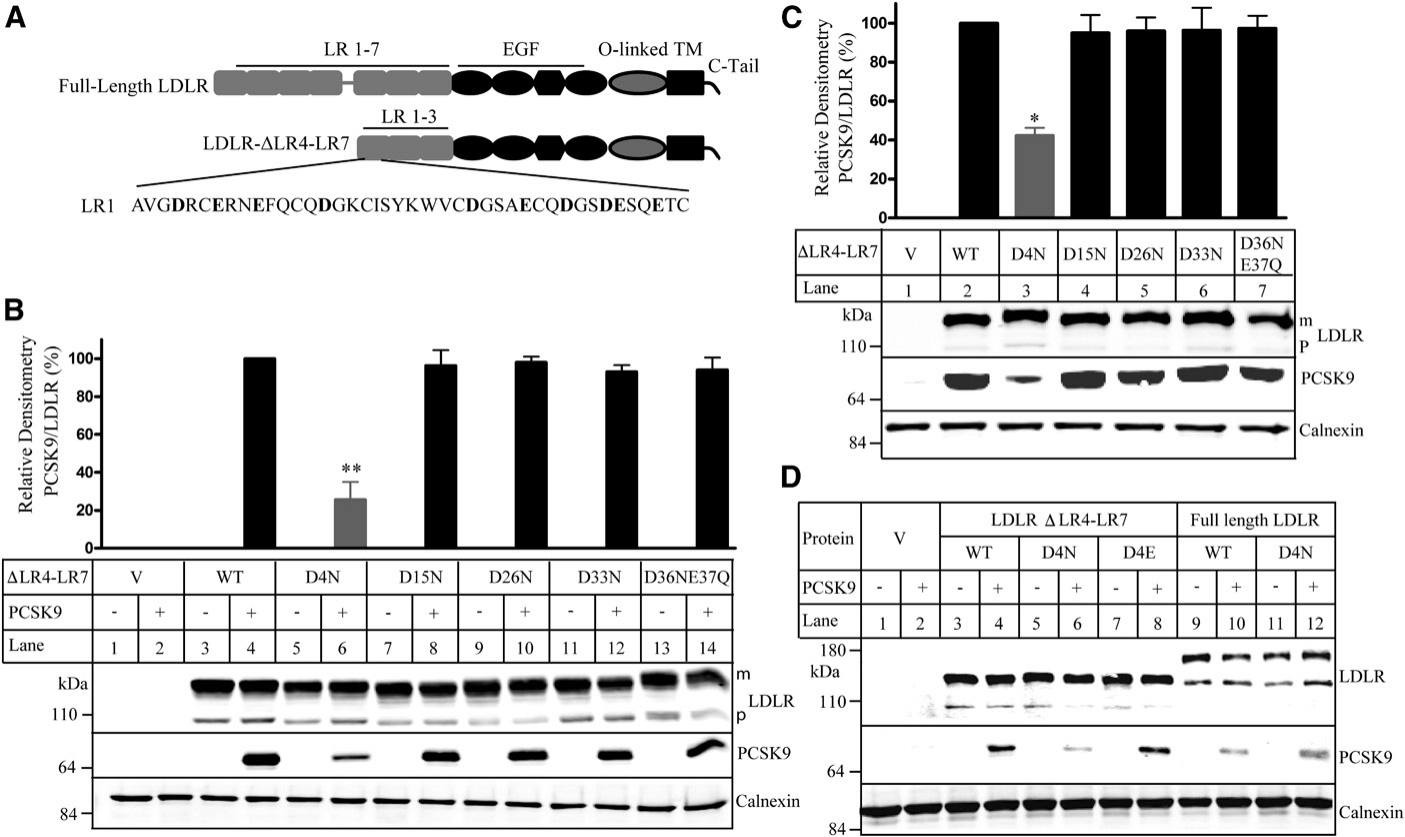
Binding of PCSK9 to the WT and mutated LDLR-ΔLR4-LR7. A: A schematic of the full-length LDLR and LDLR-ΔLR4-LR7 with an enlarged view of the LR1. Negatively charged amino acid residues in the LR1 are shown in bold. C-tail, C-terminal cytoplasmic tail; TM, transmembrane domain. B–D: Binding of PCSK9 to the WT and mutant LDLR-ΔLR4-LR7 at pH 7.4 (B) and pH 6.0 (C) or to the WT and mutant LDLR-ΔLR4-LR7 and full-length LDLR at pH 7.4 (D). HEK293 cells transiently expressing the WT or mutant LDLR-ΔLR4-LR7 or full-length LDLR were incubated with PCSK9 (2 μg/well) at pH 7.4 (B, D) or pH 6.0 (C). The same amount of whole-cell lysate was subjected to immunoblotting. The membrane was cut into halves. The top part was blotted with a monoclonal anti-LDLR Ab and a polyclonal anti-calnexin Ab. The bottom part was blotted with a monoclonal anti-PCSK9 Ab 15A6. The bar charts in B and C were a percentage of the relative densitometry PCSK9 binding signal. It was the percentage of relative densitometry of PCSK9 binding to mutant LDLR to that of PCSK9 binding to the WT LDLR that was defined as 100%. The relative densitometry of PCSK9 binding to LDLR was the ratio of the densitometry of PCSK9 to that of the mature form of LDLR. Values were mean ± SD of three or more experiments. The bottom images were representative ones of protein levels. The top bands of LDLR were the mature and fully glycosylated forms (m). The bottom bands of LDLR were the precursor forms (p). V: Cells were transfected with the empty vector, pCDNA3.1. Similar results were obtained from at least three experiments. * *P* < 0.05; ** *P* < 0.01.

The C-terminal domain of PCSK9 is positively charged, and the LRs of LDLR are negatively charged. Given the role of the LRs in PCSK9-promoted LDLR degradation (12, 21–23), we assessed whether negatively charged residues in the LRs of LDLR affected PCSK9 binding. To simplify the initial screening, we used a mutant LDLR (LDLR-ΔLR4-LR7), in which LRs 4–7 were deleted, because we have previously demonstrated that at least three LRs were required for PCSK9-promoted LDLR degradation (12). We first replaced all negatively charged residues Asp and Glu in the LR1 of LDLR-ΔLR4-LR7 with uncharged Asn and Gln individually or in combination (**Fig. 1A**). The WT and mutant receptors were transiently expressed in HEK293 cells. The cells were then prechilled on ice for 30 min to inhibit LDLR endocytosis and then incubated with or without PCSK9 on ice in a pH 7.4 buffer to mimic PCSK9 binding to LDLR at the cell surface. As shown in Fig. 1B and supplemental Fig. 1A, the expression levels of the WT and mutant LDLR-ΔLR4-LR7 were comparable in the presence and absence of PCSK9, indicating that PCSK9 did not promote LDLR degradation at the condition. After normalization to the levels of the mature form of LDLR, mutation of Asp at position 4 to Asn (D4N) significantly reduced PCSK9 binding to LDLR-ΔLR4-LR7 at pH 7.4 (Fig. 1B, lane 6 vs. 4), whereas substitution of Asp at other positions with Asn and replacement of Glu residues in the LR1 with Gln had no detectable effect on PCSK9 binding to the receptor (Fig. 1B and supplemental Fig. S1A).

Next, we performed the PCSK9 binding assay on ice in a pH 6.0 buffer to mimic PCSK9 binding at the acidic endosome as described in previous studies (21). We observed that only mutation D4N significantly reduced PCSK9 binding to the receptor in the acidic environment, whereas other mutations had no effect (Fig. 1C, lane 3 vs. 2, and supplemental Fig. S1B). We also mutated Asp4 to a similarly charged Glu (D4E) and examined PCSK9 binding to the receptor at pH 7.4. As shown in Fig. 1D, D4E mutation, unlike D4N, had no detectable effect on PCSK9 binding to LDLR-ΔLR4-LR7 (lane 8 vs. 4). Together, these findings indicate that a negative charge at position 4 of the LR1 in LDLR-ΔLR4-LR7 plays an important role in PCSK9 binding. We then replaced Asp at position 4 in the LR1 of the full-length LDLR with Asn to assess its role in PCSK9 binding. We observed that binding of PCSK9 to the WT and D4N mutant full-length LDLR at pH 7.4 was comparable (Fig. 1D, lane 12 vs. 10), indicating a negligible role of Asp at position 4 of the full-length LDLR in PCSK9 binding.

### Effect of mutations in the full-length LDLR on PCSK9 binding

**Fig. 2.**
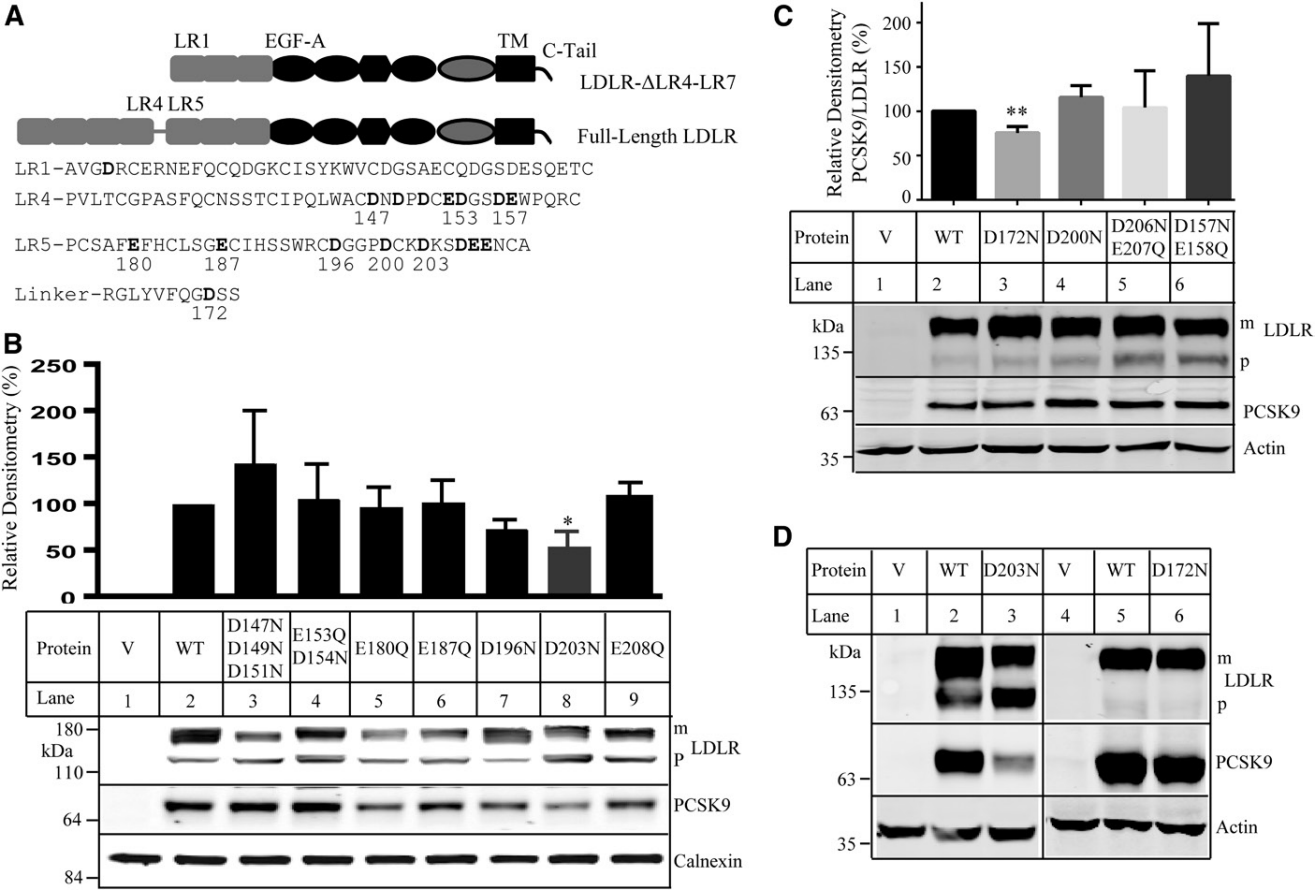
Binding of PCSK9 to the WT and mutant full-length LDLR. A: A schematic of the full-length LDLR and LDLR-ΔLR4-LR7 with an enlarged view of the LR1, LR4, LR5, and the linker. Negatively charged amino acid residues in the LR4, LR5, and the linker are shown in bold. C-tail, C-terminal cytoplasmic tail; TM, transmembrane domain. B–D: Binding of PCSK9 to the WT and mutant full-length LDLR. The experiments were performed as described in the Fig. 1 legend. Briefly, HEK293 cells transiently expressing the WT or mutant LDLR were incubated with PCSK9 (2 μg/ml) on ice at a pH 7.4 (B, C) or pH 6.0 (D) buffer as indicated above. LDLR and PCSK9 were detected by HL-1 and 15A6, respectively. The top bands of LDLR were the mature forms (m). The bottom bands of LDLR were the precursor forms (p). Data shown in the top graph in B and C were quantified as described in the Fig. 1 legend. V: Cells were transfected with the empty vector, pCDNA3.1. Values are mean ± SD of three experiments. * *P* < 0.05; ** *P* < 0.01.

Given that PCSK9 mainly binds to EGF-A of LDLR (11, 18, 20), it was possible that the relative distance of negatively charged residues in the LRs to the EGF-A affected their contributions to PCSK9 binding. The LR1 in LDLR-ΔLR4-LR7 corresponds to the LR5 in the full-length receptor (**Fig. 2A**). Considering the high flexibility of the linker region between the LR4 and LR5 (31), we replaced all negatively charged residues in the LR4, the 12-residue linker, and the LR5 of the full-length LDLR with noncharged Asn and Gln individually or in combination to examine their involvement in PCSK9 binding (Fig. 2A). We observed that, when compared with the WT LDLR, replacement of Asp at position 203 in the LR5 to Asn (D203N) reduced PCSK9 binding at pH 7.4 by approximately 50% (Fig. 2B, lane 8), and mutation of Asp at position 172 in the linker to Asn also significantly reduced PCSK9 binding at pH 7.4 (Fig. 2C, lane 3). Substitution of other negatively charged residues Asp and Glu in the LR4 and LR5 with Asn and Gln, however, had no significant effect (Fig. 2B, C). Mutation D196N had a tendency to reduce PCSK9 binding, but the reduction did not reach statistical significance (Fig. 2B, lane 7). We did not study individual amino acid residues in the combination mutations shown in Fig. 2B, C due to the lack of effect of these multiple mutations on PCSK9 binding. Next, we examined whether mutations D172N and D203N affected PCSK9 binding in the acidic endosomal environment. As shown in Fig. 2D, D203N markedly reduced PCSK9 binding at pH 6.0 (lane 3 vs. 2), whereas D172N had no detectable effect (lane 6 vs. 5). Taken together, these findings indicate that Asp172 and Asp203 play a role in PCSK9 binding to the full-length LDLR.

**Fig. 3.**
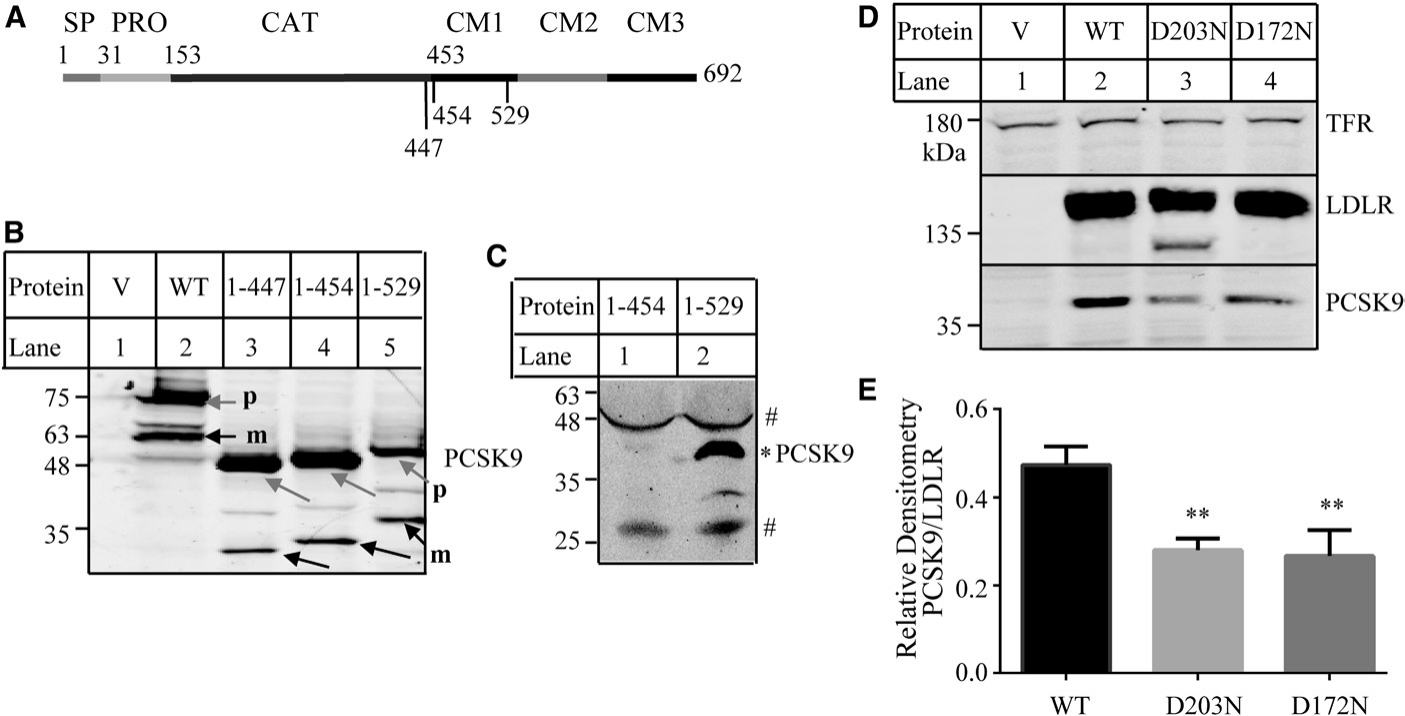
Binding of mutant PCSK9 to the WT and mutant LDLR. A: A schematic of PCSK9. Signal peptide (SP; amino acid residues 1–30); prodomain (PRO; amino acid residues 31–152); catalytical domain (CAT; amino acid residues 153–452); and C-terminal domain (amino acid residues 453–692) with three modules: module 1 (CM1; amino acid residues 457–527), module 2 (CM2; amino acid residues 534–601), and module 3 (CM3; amino acid residues 608–692) (45). B, C: Expression of the WT and mutant PCSK9 in HEK293 cells (B) and culture medium (C). HEK293 cells transiently expressing the WT or mutant PCSK9 as indicated were collected for the preparation of whole-cell lysate. Culture medium was collected from one 150 mm dish of HEK293 cells transiently transfected with mutant PCSK9^1–454^ or PCSK9^1–529^ and then concentrated using a 3 kDa cutoff centrifugal concentrator (Millipore). The same amount of total proteins of whole-cell lysate (B) or the same amount of concentrated medium (C) were subjected to immunoblotting using a monoclonal anti-PCSK9 Ab 13D3 that recognizes the catalytical domain of PCSK9. m, cleaved mature form of PCSK9 (black arrows); p, the precursor form of PCSK9 (gray arrows). # nonspecific bands; * mature form of PCSK9^1-529^. D: Binding of PCSK9^1–529^ to the WT and mutant LDLR. The experiments were performed as described in the Fig. 1 legend. Briefly, HEK293 cells transiently expressing the WT or mutant LDLR were incubated with the same amount of concentrated medium containing PCSK9^1–529^ on ice at pH 7.4 for 4 h. LDLR and PCSK9 were detected by HL-1 and 13D3, respectively. Transferrin receptor (TFR) was detected by its specific monoclonal Ab. V: Cells were transfected with the empty vector, pCDNA3.1. E: Quantified PCSK9 binding data. The relative densitometry of PCSK9 was the ratio of the densitometry of PCSK9 to that of the mature form of LDLR. Values are mean ± SD of three experiments. ** *P* < 0.01.

We then investigated the potential role of the C-terminal PCSK9 in its binding to LDLR. We made mutant PCSK9^1–447^ that lacked the whole C terminus of PCSK9 and mutant PCSK9^1–454^, in which the three C-terminal modules (amino acid residues 455–692) were deleted (**Fig. 3A**). The mutant proteins were transiently expressed in HEK293 cells and could undergo self-cleavage to produce the mature form (Fig. 3B, lanes 4 and 3, black arrows). Culture medium was collected, concentrated, and applied to immunoblotting. Neither PCSK9^1-447^ (data not shown) nor PCSK9^1–454^ (Fig. 3C, lane 1) could be detected in medium, indicating impaired secretion of the two mutant proteins. We then made mutant PCSK9^1–529^ that lacked the C-terminal modules 2 and 3 (amino acid residues 530–692) (Fig. 3A). PCSK9^1–529^ was processed in HEK293 cells to produce the mature form (Fig. 3B, lane 5, black arrow), and the mature form could be detected in the concentrated culture medium (Fig. 3C, lane 2). We then applied the same amount of concentrated medium containing PCSK9^1–529^ to HEK293 cells expressing the WT or mutant LDLR. As shown in Fig. 3D, E, both D172N and D203N significantly reduced binding of PCSK9^1-529^ to the receptor (Fig. 3D, lanes 3 and 4 vs. 2). This finding suggests that amino acid residues 530–692 in the C-terminal PCSK9 are not required for the inhibitory effect of D172N and D203N on PCSK9 binding.

**Fig. 4.**
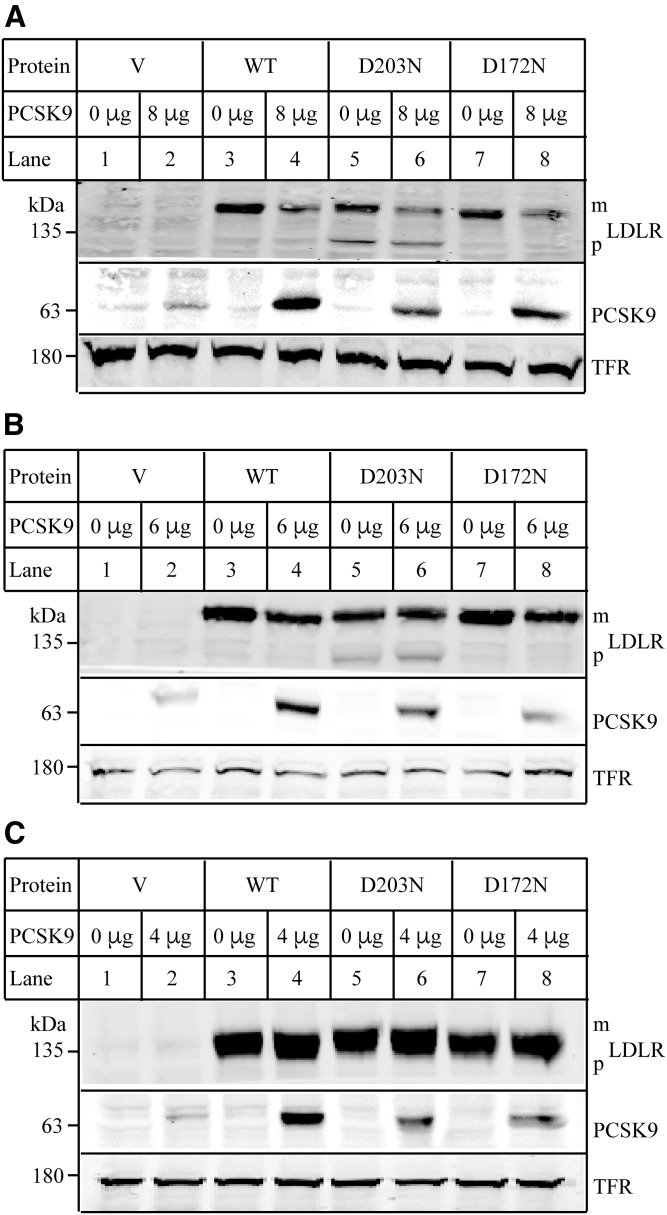
PCSK9-promoted LDLR degradation. A–C: Effects of PCSK9 on LDLR expression. HEK293 cells transiently expressing the WT or mutant LDLR were incubated with DMEM in the presence or absence of different amount of PCSK9 (A, 8 μg; B, 6 μg; and C, 4 μg) as indicated at 37°C for 12 h. After washing, whole-cell lysate was prepared, and the same amount of total proteins was subjected to immunoblotting using 15A6 (PCSK9), HL-1 (LDLR), and a monoclonal anti-transferrin receptor (TFR). Similar results were obtained from at least one more experiment.

We also examined whether mutations D172N and D203N affected PCSK9-promoted LDLR degradation. HEK293 cells expressing the WT or mutant LDLR were incubated with PCSK9 at 37°C for 12 h. We observed that PCSK9 efficiently promoted degradation of the WT and the two-mutant LDLR at a concentration of 8 μg/ml (**Fig. 4A**, lanes 4 vs. 3, 6 vs. 5, and 8 vs. 7), even though D203N- and D172N-expressing cells displayed less PCSK9 in whole-cell lysate compared with the WT LDLR-expressing cells. On the other hand, PCSK9 at a concentration of 6 μg/ml could efficiently promote degradation of the WT (Fig. 4B, lane 4 vs. 3) and mutant D172N LDLR (lane 8 vs. 7), but failed to stimulate D203N degradation (lane 6 vs. 5). However, neither the WT receptor nor the two mutant LDLRs could be efficiently degraded by addition of PCSK9 at a concentration of 4 μg/ml (Fig. 4C). These findings suggest that PCSK9-promoted degradation of D203N is less effective than its action on the WT LDLR.

### Effects of D172N and D203N on LDL binding and LDLR trafficking

**Fig. 5.**
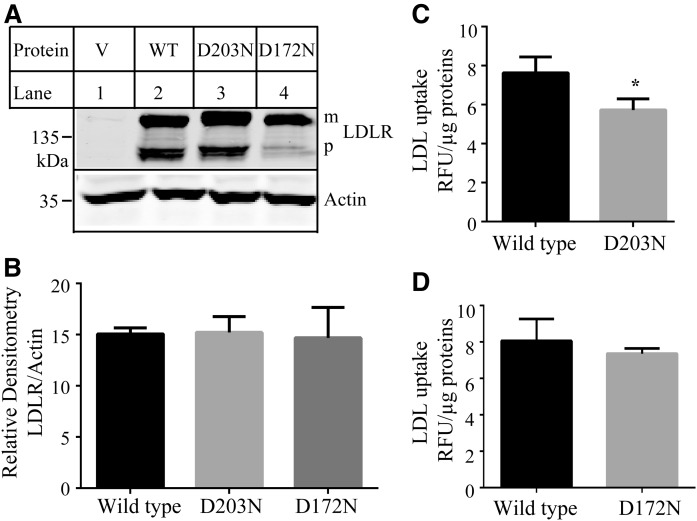
Binding of LDL to the WT and mutant LDLR. A, B: Expression of the WT and mutant LDLR in Hepa1c1c7 cells. Briefly, the same amount of total proteins isolated from Hepa1c1c7 cells transiently expressing the WT or mutant LDLR were subjected to immunoblotting using a monoclonal anti-LDLR Ab, HL-1, and a monoclonal anti-actin Ab. The relative densitometry of LDLR was the ratio of the densitometry of LDLR to that of actin. Values were mean ± SD of three experiments. C, D: LDL uptake. Briefly, Hepa1c1c7 cells transiently expressing the WT or mutant LDLR were incubated with Dil-LDL in the presence or absence of LDL. After washing, the fluorescence signal was measured. The relative fluorescence units (RFU) were normalized to total proteins (micrograms). The amount of specific LDL uptake was the difference between the total counts measured in the absence of unlabeled LDL and the counts measured in the presence of an excess of unlabeled LDL (nonspecific background fluorescence).

LDLR directly binds to LDL and mediates cellular LDL uptake. Thus, we investigated whether the two mutations that impaired PCSK9 bindings affected the ability of the receptor to bind LDL using Dil-LDL as described (28). We found that the levels of D203N and D172N in Hepa1c1c7 cells were comparable to that of the WT receptor (**Fig. 5A**, B), but D203N significantly reduced LDL binding (Fig. 5C), whereas D172N showed a similar LDL binding capacity as the WT LDLR (Fig. 5D).

**Fig. 6.**
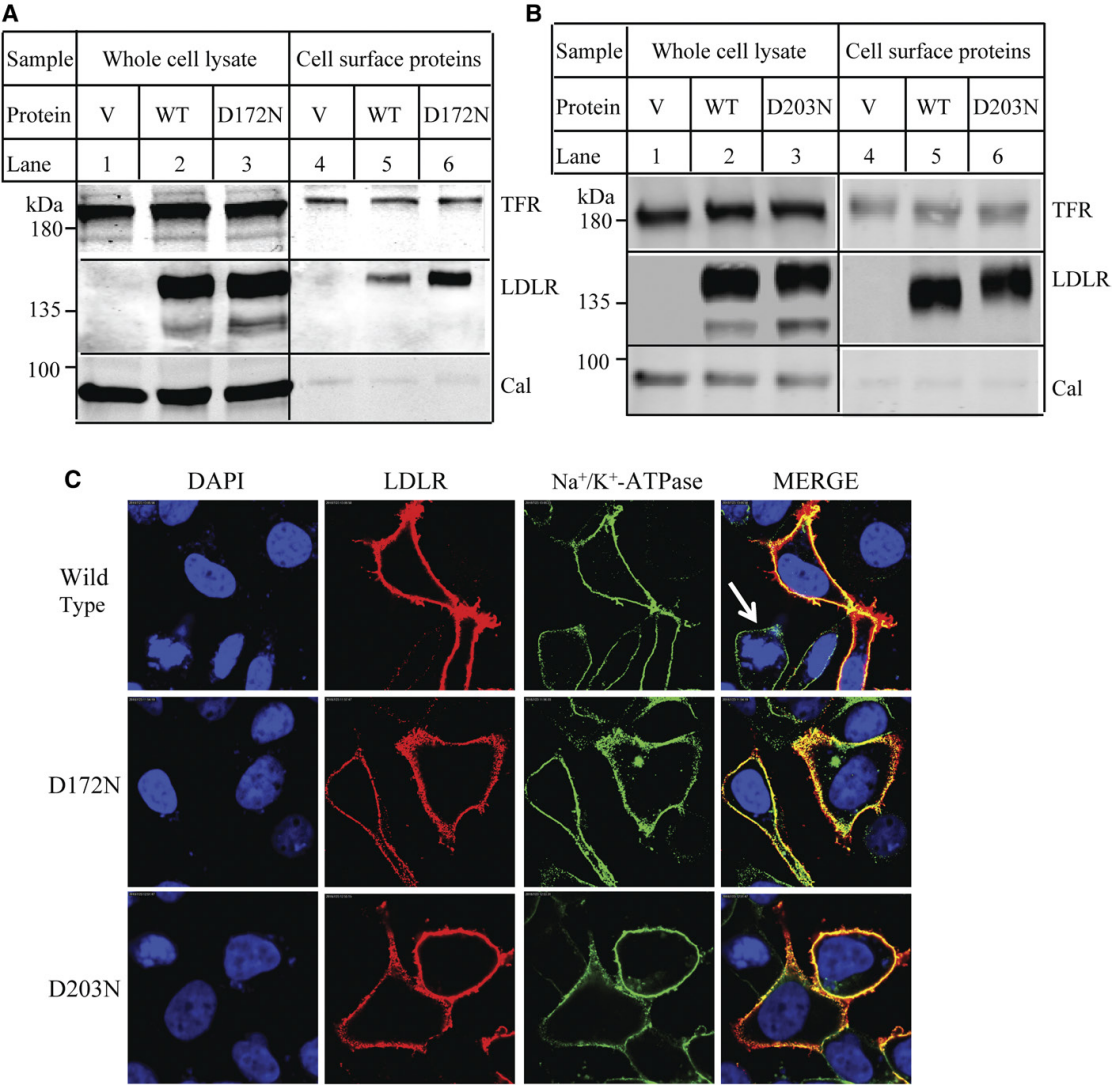
Cellular localization of the WT and mutant LDLR. A, B: Biotinylation of cell surface proteins. HEK293 cells transiently expressing the WT or mutant LDLR were incubated with Sulfo-(LC)-NHS-biotin. The whole-cell lysate was then prepared and subjected to Neutravidin agarose to pull down biotinylated cell surface proteins. LDLR was detected by HL-1. Calnexin (Cal) and transferrin receptor (TFR) were detected by their specific Abs. V: Cells were transfected with the empty vector, pCDNA3.1. C: Confocal microscopy. HEK293 cells transiently expressing the WT or mutant LDLR were fixed, permeabilized, and then incubated with a monoclonal anti-LDLR Ab and a polyclonal anti-Na^+^-K^+^-ATPase Ab. Ab binding was visualized with Alexa 568-conjugated goat anti-mouse IgG (red) and Alexa 488-conjugated goat anti-rabbit IgG (green). Nuclei were visualized with DAPI and shown as blue. An *x*-*y* optical section of the cells illustrates the distribution of the WT and mutant proteins between plasma and intracellular membranes (magnification: 100×).

Binding of PCSK9 and LDL to the receptor mainly occurs at the cell surface. To determine whether the effect of D172N and D203N on PCSK9 binding might be attributable in part to changes in trafficking of LDLR, we labeled cells with biotin and precipitated biotinylated surface proteins from whole-cell lysate using Neutravidin agarose. As shown in **Fig. 6A**, B, the ratio of the cell-surface levels of the two mutants, D172N and D203N, to their levels in whole-cell lysate was comparable to that of the WT LDLR, indicating that the two mutants could be transported to plasma membrane. To further confirm these findings, we monitored the localization of the mutant as well as the WT LDLR in HEK293 cells by confocal microscopy. LDLR was shown as red. The plasma membrane marker, Na^+^/K^+^-ATPase, was shown as green. Endogenous levels of LDLR in HEK293 were very low. Thus, the LDLR signal was undetectable in nontransfected cells (Fig. 6C, arrow in top merged panel). We found that a majority of the WT LDLR could be detected on the cell periphery (top panel), which was colocalized with Na^+^/K^+^-ATPase that was shown as yellow in the merged panel (top panel, right). D172N (middle panel) and D203N (bottom panel) were also colocalized with Na^+^/K^+^-ATPase, indicating that the two mutant LDLRs could be transported to the plasma membrane in HEK293 cells.

### Effect of mutations of Asp172 and Asp203 on PCSK9 binding

**Fig. 7.**
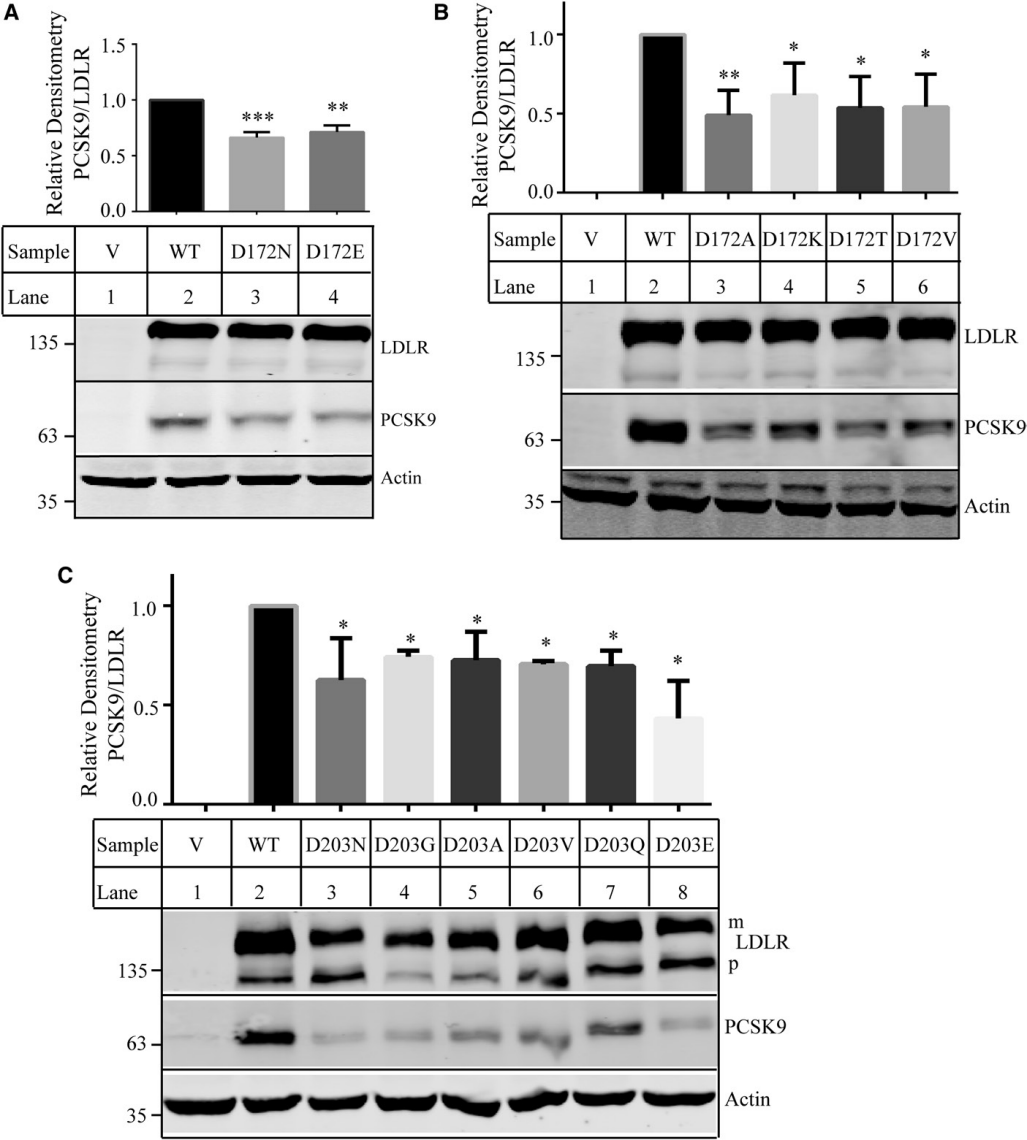
Binding of PCSK9 to the WT and mutant LDLR. A–C: Effects of mutations of Asp172 (A, B) and Asp203 (C) on PCSK9 binding. The experiments were performed as described in the Fig. 1 legend. Briefly, HEK293 cells transiently expressing the WT or mutant LDLR were incubated with PCSK9 (2 μg/well) at pH 7.4 for 2 h on ice. LDLR and PCSK9 were detected by HL-1 and 15A6, respectively. Actin was detected by a monoclonal Ab. The bottom images were representative ones of protein levels. The top bar chart was a percentage of the relative densitometry of PCSK9 to that of the mature form of LDLR. It was calculated as described in the Fig. 3E legend. V: Cells were transfected with the empty vector, pCDNA3.1. Values were mean ± SD of three or more experiments. * *P* < 0.05; ** *P* < 0.01; *** *P* < 0.005.

Next, we investigated how specific the requirements were for Asp at positions 172 and 203 of LDLR for its ability to bind PCSK9 at pH 7.4. First, we replaced Asp172 and Asp203 with Glu (D172E and D203E) to determine whether another acidic residue could substitute for Asp. Asp172 was also mutated to Ala, Lys, Thr, and Val. Asp203 was replaced with Gly, Ala, Val, and Gln. The WT and mutant LDLR were transiently expressed in HEK293 cells, and the PCSK9 binding assay was performed at pH 7.4. After normalization for differences in the levels of the mature form of LDLR, we found that mutation of Asp172 to another negatively charged residue Glu or other residues (Ala, Lys, Thr, or Val) all significantly reduced the ability of LDLR to bind PCSK9 (**Fig. 7A**, B). All mutations at position 203 that we tested, including D203E, also significantly decreased PCSK9 binding (Fig. 7C). Thus, it appears that an Asp residue at positions 172 and 203 is specifically required for efficient PCSK9 binding.

We noticed that mutation D203N slightly increased the molecular mass of LDLR compared with that of the WT receptor (Fig. 2B, lane 8; Fig. 2D, lane 3; and Fig. 6B, lanes 3 and 6). Considering that D203N introduces a novel canonical *N*-glycosylation motif (Asn-X-Ser) to the receptor (supplemental Fig. S2A), we investigated whether the mutation conferred an extra *N*-glycan to LDLR. Whole-cell lysate isolated from the WT and mutant LDLR-expressing cells was treated with PNGase F that cleaves oligosaccharides from the side-chain amide of Asn. LDLR contains two *N*-linked oligosaccharides (32). Indeed, PNGase F treatment reduced the molecular mass of the WT LDLR, indicating that the enzyme efficiently removed *N*-linked glycans from the receptor (supplemental Fig. S2B, lane 5 vs. 2). The size of mutant D203N was also reduced (lane 6 vs. 3) and comparable to that of the WT LDLR when treated with PNGase F (lane 6 vs. 5), indicating that PNGase F eliminated the increase in the molecular mass of mutation D203N. Next, we treated cells with tunicamycin that specifically inhibits *N*-linked glycosylation. LDLR contains only two *N*-glycosylation sites and is mainly *O*-glycosylated at the side chains of Ser and Thr residues (32). Consistent with previous reports (33), tunicamycin slightly reduced the molecular mass of the mature and precursor forms of the WT LDLR (supplemental Fig. S2C, lane 4 vs. 3) because protein glycosylation starts in the ER and is matured in the Golgi. The molecular mass of the precursor of mutant D203N was also reduced by inhibition of *N*-linked glycosylation and displaced a similar size as the precursor of the WT LDLR (lane 6 vs. 4). However, tunicamycin essentially eliminated the mature form of D203N without detectable effect on that of the WT receptor (supplemental Fig. S2C, lane 6 vs. 4). The amount of the mature form of D203G and D203V was also reduced by tunicamycin, but to a lesser extent than that of D203N. These findings indicate that mutation D203N might introduce an extra *N*-glycan to LDLR.

## DISCUSSION

In the current study, we demonstrated the important role of negatively charged amino acids in the LRs of LDLR in PCSK9 binding. We showed that elimination of the negative charge on Asp at position 4 in the LR1 of mutant LDLR-ΔLR4-LR7 significantly reduced PCSK9 binding, whereas replacement of Asp4 in the full-length LDLR with Asn had no detectable effect. On the other hand, mutations D172N in the linker between the LR4 and LR5 and D203N in the LR5 of the full-length LDLR significantly reduced PCSK9 binding to the receptor at pH 7.4. Furthermore, D203N, but not D172N, reduced PCSK9 binding at pH 6.0 and LDLR-mediated LDL uptake. Together, these findings indicate that Asp residues at specific positions in the LRs regulate binding of PCSK9 to LDLR.

It has been reported that the side chain of Asp203 forms hydrogen bonds with the backbone amide of the LR5 (34), thereby playing an important role in maintaining structural stability. Indeed, mutations on Asp203 including D203N, D203G, D203A, and D203V have been identified in FH patients (35–37). However, we did observe the mature form and cell-surface expression of D203N, indicating that the mutant LDLR could be delivered to plasma membrane. When normalized to the mature form of LDLR, D203N significantly reduced binding of PCSK9 and LDL to the receptor and PCSK9-promoted LDLR degradation. It is of note that patients carrying D203N display FH. Thus, the effect of D203N on LDL binding must be dominant over its effect on PCSK9 binding. In addition, mutations D203N, D154N, and D172N all introduced a novel *N*-glycosylation site to the receptor (supplemental Fig. S2A). D203N and double-mutation E153QD154N, but not D172N, seemed to cause a minor electrophoretic mobility shift on SDS-PAGE (Fig. 2B, C). However, E153QD154N, unlike D172N and D203N, had no detectable effect on PCSK9 binding. In addition, we found that PNGase F reduced the gel shift caused by D203N, whereas tunicamycin treatment essentially eliminated the mature form of D203N, but had much less effect on trafficking of other D203 mutations. It would be of interest to investigate whether and how these mutations affect *N*-glycosylation of LDLR and the related functional consequences.

Asp172 resides in the highly flexible C-terminal half of the linker between the LR4 and LR5 (31). No FH mutation has been reported at this position, except for a frameshift mutation that deletes three amino acid residues (172–174) and causes a premature stop (38). We found that mutation D172N showed a similar distribution pattern as the WT receptor in the Western blot and confocal microscopy. Furthermore, mutation D172N had no detectable effect on LDL binding at pH 7.4 and PCSK9 binding at pH 6.0. These strongly suggest that D172N does not cause a major perturbation of the structure of the protein. However, we cannot exclude the possibility that D172N may result in a subtle structural change in LDLR.

PCSK9 mainly binds to the EGF-A domain of LDLR. How did mutations D172N and D203N in the ligand-binding regions impair PCSK9 binding to the receptor? The crystallography structure of PCSK9 with the complete extracellular domain of LDLR at an acidic or neutral value is currently unavailable. Thus, how exactly D172N and D203N affect PCSK9 binding, especially at a neutral pH (cell surface), is unclear. We previously found that at least three LRs were required for PCSK9-promoted LDLR degradation (12). Tyeten et al. (21) also reported that binding of PCSK9 to mutant LDLR without the LRs was significantly lower at a neutral or acidic pH value than its binding to the full-length receptor. Yamamoto et al. (23) further demonstrated that the LRs of LDLR bound to C-terminal PCSK9 at a pH-dependent manner with much stronger binding in the acidic endosomal environment. In addition, studies from Holla et al. (22) indicated that the positively charged C-terminal PCSK9 might interact with the negatively charged LRs of LDLR. Similarly, Cunningham et al. (39) used the surface plasmon resonance assay and revealed that C-terminal PCSK9 purified from CHO cells was important for PCSK9 binding to LDLR. These findings indicate that the negatively charged LRs of LDLR may interact with the positively charged C-terminal PCSK9. In this situation, D203N might affect the structural stability of the LR5 of LDLR and consequently impair PCSK9 binding. On the other hand, Asp172 that only affected PCSK9 binding at the neutral pH might involve the interaction with PCSK9. Further mutational analysis studies revealed that replacement of Asp at position 172 or 203 with other residues tested, including the negatively charged Glu, all significantly reduced PCSK9 binding, indicating a specific requirement of Asp at these two positions. It is possible that both the negative charge and the length of the side chain of amino acid residues at positions 172 and 203 are important for PCSK9 binding. In addition, we found that both D203N and D172N significantly reduced binding of mutant PCSK9^1-529^ to the receptor. This suggests that positively charged residues located between amino acids 529 and 692 in the C-terminal PCSK9 are not required for the effect of D203N and D172N on PCSK9 binding. Removal of the complete three C-terminal modules (amino acid residues 454–692), however, dramatically impaired PCSK9 secretion. Studies are ongoing in the laboratory to define the minimum length of C-terminal PCSK9 required for its efficient secretion and the potential impact of amino acid residues 454–529 in PCSK9 on its binding to LDLR.

Findings discussed above were based on results obtained from cultured cell-based binding assays and PCSK9 purified from mammalian cells. On the other hand, two other groups, Bottomley et al. (19) and Lo Surdo et al. (20), reported that the full-length and C-terminal deletion PCSK9 purified from *Escherichia coli* bound to LDLR with a similar binding capacity. Lo Surdo et al. (20) proposed that the entire LRs of LDLR did not directly associate with PCSK9 at a neutral pH value. Based on this model, the two Asp residues we identified would not directly interact with PCSK9 at pH 7.4. Given the flexibility of the loops between the LRs (5, 20, 31), it is possible that the structural alteration in the LR5 caused by D203N may affect the structural stability of the LR7 via a possible intermodule cross-talk between different LRs, as reported by Guttman and Komives (31). This may eventually impair the integrity of the EGF-A due to the presence of the rigid structure between the LR7 and the EGF-A (40) and consequently interfere with PCSK9 binding to the receptor. However, this cannot explain why mutation D172N impaired PCSK9 binding only at pH 7.4. In addition, mutations of other negatively charged residues in the LR4 and LR5, including FH mutations D147N, D151N, D154N, and D157N that are believed to cause damaging effects on LDLR structure (41) and mutations of Asp at positions 196, 200, and 206, and Glu at position 207 that disrupt the calcium binding pocket of LR5 and its structure integrity (34, 42), had no detectable effect on PCSK9 binding. Thus, how exactly D172N and D203N affect PCSK9 binding is unclear.

We noticed that, in the Bottomley et al. study (19), the apparent IC_50_ value for mutant PCSK9 without the C terminus to suppress the PCSK9-LDLR interaction was approximate 110 nM, whereas it was 80 nM for the full-length PCSK9. Similarly, the binding affinity of the extracellular domain of LDLR without the LR1-LR6 for PCSK9 was slightly lower than that of the complete extracellular domain of LDLR at pH 7.4 (*K*_d_, 880 vs. 750 nM) (20). We also observed that mutations D172N and D203N only mildly reduced PCSK9 binding. Together, these findings indicate a noncritical role of the LR1-LR6 of LDLR in PCSK9 binding. However, it is also possible that the LRs, like the YWTD domain shown in the crystal structure (20), may have a minor interaction with PCSK9. Alternatively, several PCSK9 potential binding partners have been reported (14, 43, 44). It will be of interest to investigate whether the LRs of LDLR, especially D172 and D203, are involved in the interaction with these potential partners.

## Supplementary Material

Supplemental Data
