# Supplementary material for: Identification of amino acid residues in the ligand binding repeats of LDL receptor important for PCSK9 binding
Source: J Lipid Res. 2019 Jan 7;60(3):516–27. doi: 10.1194/jlr.M089193 (PMC6399494; doi:10.1194/jlr.M089193)
Supplement: Supplemental Data [file supp_60_3_516__index.html]

Identification of amino acid residues in the ligand binding repeats of LDLR important for PCSK9 binding — Identification of amino acid residues in the ligand binding repeats of LDL receptor important for PCSK9 binding — Supplemental Data 

# Identification of amino acid residues in the ligand binding repeats of LDL receptor important for PCSK9 binding

## Supplemental Data

- Supplemental Figure S1 and S2 (.pdf, 1.6 MB) - Supplemental Figure S1. Binding of PCSK9 to the wild-type and mutant LDLR-??LR4-LR7 LDLR at pH 7.4 (A) or pH 6.0 (B). Supplemental Figure S2. Effect of mutation D203N on LDLR expression.
