# Supplementary material for: Identification of amino acid residues in the ligand binding repeats of LDL receptor important for PCSK9 binding
Source: J Lipid Res. 2019 Jan 7;60(3):516–27. doi: 10.1194/jlr.M089193 (PMC6399494; doi:10.1194/jlr.M089193)
Supplement: Supplemental Data [file 10.1194_M089193_jlr.M089193-1.pdf]

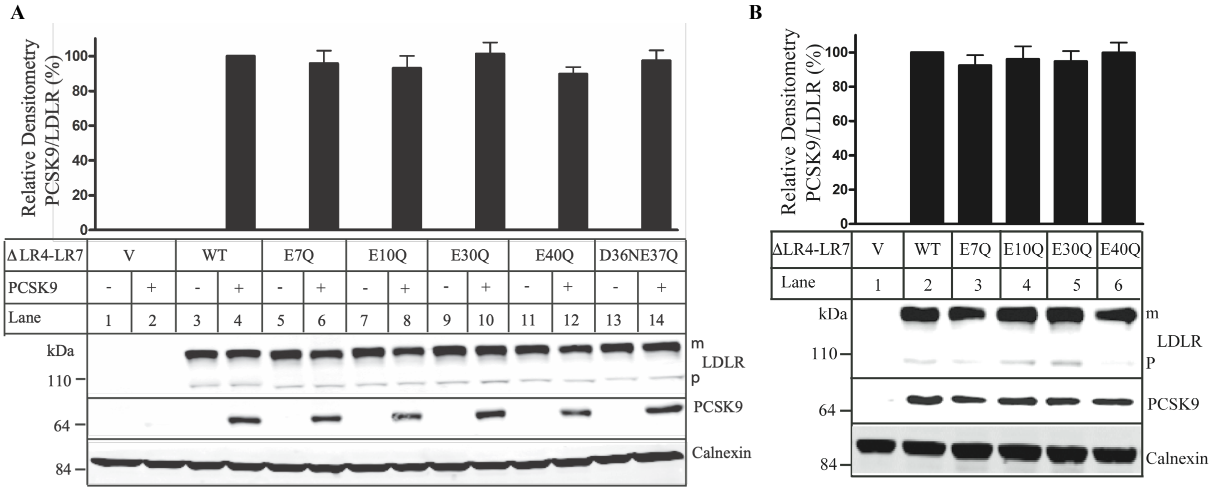

Supplemental Figure S1. Binding of PCSK9 to the wild-type and mutant LDLR-ΔLR4-LR7 LDLR at pH 7.4 (A) or pH 6.0 (B). The experiments were performed as described in the legend to Figure 1. LDLR and PCSK9 were detected by HL-1, and 15A6, respectively. Calnexin was detected by a polyclonal antibody. Top bands of LDLR were the mature and fully glycosylated forms (m). Bottom bands of LDLR were the precursor forms (p). Bottom figures were representative ones of protein levels. V: cells were transfected with the empty vector, pCDNA3.1. Data shown in top figures were quantified as described in the legend to Figure 1B. The percentage of the relative densitometry of PCSK9 binding to the wild-type LDLR was defined as 100%. Values were mean ± S.D. of ≥ 3 experiments.

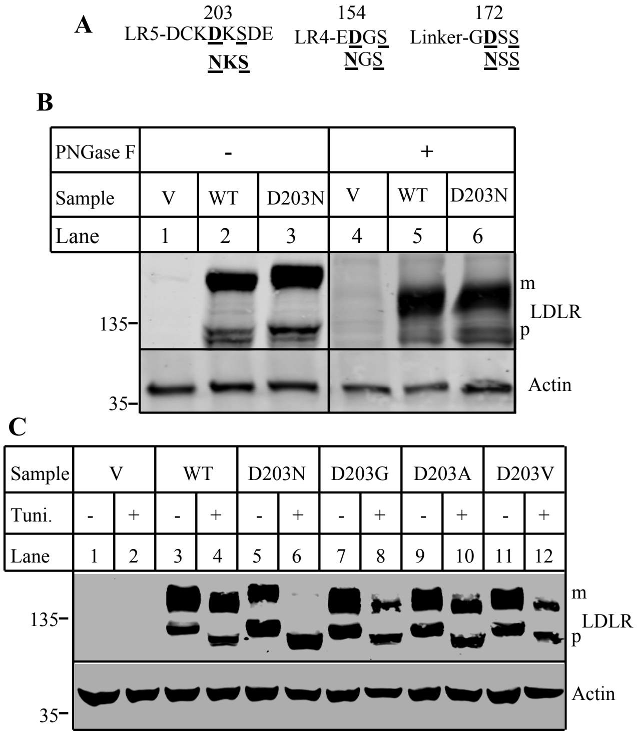

Supplemental Figure S2. Effect of mutation D203N on LDLR expression. ***A.*** Amino acid sequences showing the canonical N-glycosylation motif introduced by mutations D203N, D154N, and D172N (underlined and bold). ***B.*** Deglycosylation of proteins in whole cell lysate using PNGaseF. HEK293 cells transiently expressing the wild-type (WT) or mutant LDLR (D203N) were collected for the preparation of whole cell lysate. Same amount of total proteins was incubated with or without PNGaseF and then subjected to SDS-PAGE (5%) and immunoblotting. ***C***, Inhibition of N-glycosylation of proteins using tunicamycin. HEK293 cells transiently expressing the wild-type (WT) or mutant LDLR were incubated with tunicamycin (0.5 µg/ml) for 24 h. After, whole cell lysate was prepared and equal amount of total proteins was analyzed by immunoblotting. LDLR and actin were detected by HL-1 and a polyclonal anti-actin antibody, respectively. Top bands of LDLR were the mature forms (m). Bottom bands of LDLR were the precursor forms (p). V: cells were transfected with the empty vector, pCDNA3.1. Similar results were obtained from at least one more experiment.
